# Concurrent analysis of hospital stay durations and mortality of emerging severe acute respiratory coronavirus virus 2 (SARS-CoV-2) variants using real-time electronic health record data at a large German university hospital

**DOI:** 10.1017/ash.2023.153

**Published:** 2023-05-04

**Authors:** Derek Y. Hazard, Marlon Grodd, Adeline Makoudjou, Sara Lozano, Andrea Prunotto, Patric Tippmann, Daniela Zöller, Philipp Mathé, Siegbert Rieg, Martin Wolkewitz

**Affiliations:** 1 Institute of Medical Biometry and Statistics, Faculty of Medicine and Medical Center, University of Freiburg, Freiburg, Germany; 2 Division of Infectious Diseases, Department of Medicine II, Faculty of Medicine and Medical Center, University of Freiburg, Freiburg, Germany

## Abstract

Multistate methodology proves effective in analyzing hospitalized coronavirus disease 2019 (COVID-19) patients with emerging variants in real time. An analysis of 2,548 admissions in Freiburg, Germany, showed reduced severity over time in terms of shorter hospital stays and higher discharge rates when comparing more recent phases with earlier phases of the pandemic.

Predicting the clinical progress of hospitalized coronavirus disease 2019 (COVID-19) patients in the face of emerging variants is a crucial challenge for the medical community. This undertaking has far-reaching consequences for not only the determination of high-risk groups for the disease in a dynamic situation but also the efficient allocation of medical resources (especially when they are scarce). We sought to estimate the duration of hospital stay and in-hospital mortality of COVID-19, analyses that are vulnerable to severe competing risks and time-dependent biases^1^ that can invalidate traditional methods of estimation. Furthermore, we sought to avert the selection bias that occurs when current cases are removed from the analysis; a bias that impaired some reports at the outset of the pandemic.^2^

## Methods

Data from patients with a hospital admission 7 days before or 14 days after a positive severe acute respiratory coronavirus virus 2 (SARS-CoV-2) test were extracted from electronic health records (EHRs). Cases were categorized by sex, age, and phases of the pandemic based on the predominant variants (sequenced samples >50%) as published by the German health authorities.^3^ The following periods were used as surrogate variables for the variants and treatment options in the specified calendar period. January 27, 2020, to February 14, 2021, was characterized by SARS-CoV-2 wild-type dominance, with possible treatments with dexamethasone and remdesivir as well as minimal vaccination. February 15, 2021, to June 20, 2021, was characterized by SARS-CoV-2 α (alpha) variant dominance, with initial antibody therapy availability and vaccination of high-risk groups. June 21, 2021, to December 26, 2021, was characterized by SARS-CoV-2 δ (delta) variant dominance, with the availability of inpatient intravenous antiviral therapy and vaccination of the general population as well as booster vaccinations for high-risk groups. Finally, December 27, 2021, to March 1, 2022, was characterized by SARS-CoV-2 ο (omicron) variant dominance, with additional oral antiviral therapy availability and booster vaccination of the general population.

We employed a multistate model to estimate the durations of hospital stays and mortality as described previously^4^ with 2 transitory states (ie, nonsevere, related to regular ward admission, and severe, related to intensive care unit admission)^5^ and 2 terminal states (in-hospital death and discharge alive). In addition, we performed multivariable Cox regression on the competing terminal states.

## Results

For the entire cohort at day 30 after admission, the predicted mortality was 9.3%; 5.1% were predicted to have severe illness, and 1.4% were predicted to have nonsevere illness. Also, 84.1% were predicted to be discharged alive at day 30 (Supplementary Material 1). The total expected length of hospital stay at day 30 for the full cohort was 8.62 days (95% CI, 7.95–9.28), of which 5.09 days were estimated for nonsevere illness plus 3.52 days for severe illness.

**Fig. 1.**
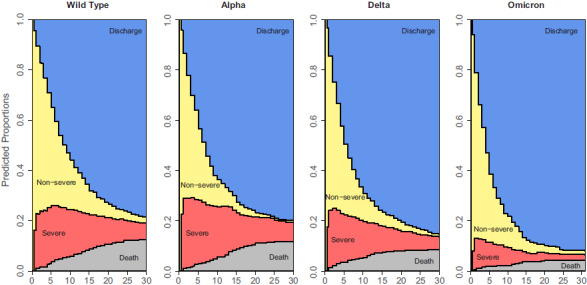
Stacked probability plots stratified by pandemic phases of 2,548 COVID-19 admissions at the University Medical Center Freiburg, Germany. SARS-CoV-2 wild-type period, January 27, 2020–February 14, 2021; SARS-CoV-2 α (alpha) period, February 15, 2021–June 20, 2021; SARS-CoV-2 δ (delta) period, June 21, 2021–December 26, 2021; SARS-CoV-2 ο (omicron) period, December 27, 2021–March 1, 2022. Calendar periods are a surrogate for the variants and treatment options in the specified period. Distance between lines represent the proportion of patients in a particular state on day since hospital admission. Note. Discharge, discharged alive from hospital; nonsevere, normal hospital ward; severe, intensive care unit; death, death in hospital.

The calendar comparison revealed a decreasing expected length of stay with each subsequent phase. This trend is illustrated in the stacked probability plots shown in Figure 1 (see Supplementary Material 3 and 4 for sex and age category). The first 2 phases displayed the highest predicted death probability on day 30. Multivariable Cox results are provided in Table 1. After adjusting for sex and age category, the SARS-CoV-2 α (alpha) phase had the highest mortality hazard ratio (1.40; 95% CI, 1.03–1.90).

**Table 1.** Results From 2,548 Admissions for COVID-19 in Freiburg, Germany

Variable	Group Size, No. (%)	Total ELOS at day 30 After Admission, Days (95% CI)	Severe State ELOS at Day 30 After Admission, Days (95% CI)	Hospital Death, aHR (95% CI)	Hospital Discharge Alive, aHR (95% CI)
**Sex**					
Female	1,150 (45.1)	7.9 (7.0–8.9)	2.8 (2.2–3.4)	Reference	Reference
Male	1,398 (54.9)	9.2 (8.3–10.0)	4.1 (3.6–4.7)	1.47 (1.11–1.95)	0.88 (0.81–0.96)
**Age group**					
0–11 y	208 (8.2)	4.5 (3.2–6.0)	1.4 (0.6–2.3)	0.96 (0.33–2.81)	1.62 (1.38–1.90)
12–25 y	192 (7.5)	5.1 (3.8–6.6)	1.2 (0.5 -2.0)	0.57 (0.17–1.89)	1.51 (1.27–1.80)
26–49 y	610 (23.9)	7.6 (6.4–8.6)	3.3 (2.6–3.9)	Reference	Reference
50–69 y	923(36.2)	10.3 (9.1–11.5)	5.5 (4.8–6.3)	2.01 (1.30–3.10)	0.74 (0.66–0.84)
70–79 y	348 (13.7)	9.9 (8.3–11.6)	3.6 (2.7–4.5)	2.73 (1.70–4.38)	0.74 (0.64–0.87)
≥80 y	267 (10.5)	9.5 (8.2–11.0)	0.8 (0.4–1.2)	3.40 (2.06–5.61)	0.77 (0.66–0.90)
**Pandemic SARS-CoV-2 phase**					
Wild type	783 (30.7)	10.4 (9.3–11.5)	4.3 (3.6–4.9)	Reference	Reference
Alpha (α)	434 (17.0)	9.2 (7.8–10.7)	4.8 (3.9–5.8)	1.40 (1.03–1.90)	1.12 (0.99–1.28)
Delta (δ)	733 (28.8)	8.2 (7.2–9.3)	3.5 (3.0–4.2)	1.11 (0.81–1.51)	1.24 (1.11–1.38)
Omicron (ο)	598 (23.5)	6.4 (5.5–7.3)	1.6 (1.2–2.1)	0.70 (0.43–1.10)	1.56 (1.39–1.76)

Note. ELOS, expected length of stay; aHR, adjusted hazard ratio; CI, confidence interval calculated with robust standard errors; SARS-CoV-2 wild-type period, January 27, 2020–February 14, 2021; SARS-CoV-2 α (alpha) period, February 15, 2021–June 20, 2021; SARS-CoV-2 δ (delta) period, June 21, 2021–December 26, 2021; SARS-CoV-2 ο (omicron) period, December 27, 2021–March 1, 2022. Periods are a surrogate for the variants and treatment options in the specified calendar period.

## Discussion

We calculated duration of stay, etiological, and risk estimates from a large data set of hospitalized COVID-19 patients based on multistate methodology avoiding many pitfalls. The strength of multistate models is highlighted in their divergence from their traditional, yet biased alternatives. A naive (ie, not accounting for competing risks) Kaplan-Meier estimate would have shown a 30-day survival probability of 72% based on the full cohort. This finding contrasts with a survival probability of 91% from the multistate model: a massive 19% difference.

Although median length-of-stay estimates have been provided in many publications,^6^ these estimates are invalid when there is a high degree of censoring. Notably, a median length of hospital stay for severely ill patients of 13 days could be determined by ignoring the time dependency of patients transitioning into severe illness. This naive estimate is based on knowing at admission which patients will move into the severe state (an impossible proposition) and does not give information on when or for how long they will remain in this state. The multistate result of an expected 3.5 days of severe illness (95% CI, 3.1–3.9) conditioned on patients being admitted as nonsevere on day 0 is not grounded in knowing the future and is far more informative. ‘Severe’ and ‘nonsevere’ illness have starkly different consequences in terms of costs and resources. In further contrast to traditional methods, the multistate analysis can be extended to condition on different days and states (Supplementary Material 4).

Improper treatment of current cases (by removing them from the data set) would have resulted in an adjusted odds ratio of 0.81 (95% CI, 0.59–1.14) to be discharged alive for patients in the SARS-CoV-2 ο (omicron) phase using logistic regression. This biased point estimate implies that patients in the SARS-CoV-2 ο (omicron) phase had a lower odds of being discharged alive, contrary to the properly calculated hazard ratio 1.56 (95% CI, 1.39–1.76) using Cox regression. These patients, in fact, had a higher discharge rate.

The interplay among the duration estimates, plots, and regression results is illustrative of why all are needed to attain a full account. The expected lengths of stay in the initial pandemic phase were longer than for the 3 subsequent phases. Analyzing a stacked probability plot, we observed a higher predicted death risk during the SARS-CoV-2 α (alpha) period. The influence of death would imply a shorter length of stay. However, we did not observe the higher death risk in the SARS-CoV-2 δ (delta) and SARS-CoV-2 ο (omicron) periods. In the last 2 phases, the opposing force of discharge alive (ie, quantified in the significantly higher hazard ratios) resulted in shorter hospital stays. Moreover, determining whether discharge alive (clinical improvement) or death (clinical failure) causes shorter stays is crucial for treatment comparisons.^7^

It is a strength that the proposed methods can be conducted in real time, thus giving an immediate impression of changing circumstances. The use of EHRs is also advantageous in that extraction could be standardized to allow data conglomeration and meta-analyses. Although the positive-result time window facilitates this standardization, it is likely that a good proportion of patients were admitted for reasons other than COVID-19.^8^ Further bias may have been introduced through EHR coding limitations at the outset and varying testing guidelines throughout the studied periods. The analysis was also limited by the absence of adjustment for other potential confounders such as issues of delayed care, specific treatments, or prior infection. Moreover, caution must be taken in extrapolating to other geographic regions where “severe” may have different interpretations. Lastly, calendar periods served as surrogate variables for the predominant variants, and claims have not been made regarding their intrinsic disease severity.

Alternative multistate methods to those presented might also be explored. Parametric models^9^ can extrapolate outside the time frame in which the data were collected, overcoming a shortcoming of nonparametric estimators. Despite not predicting the progression of patients as demonstrated here, length-of-stay estimates conditioned on the final pathway of patients can be used for resource planning.^10^

Without ignoring the seriousness of the COVID-19 pandemic, we should learn as much as we can from this crisis. As demonstrated here, multistate methodology should be used in the analyses of future patients, whether for COVID-19 or subsequent pandemics.

## Supplementary material

For supplementary material accompanying this paper visit http://doi.org/10.1017/ash.2023.153. click here to view supplementary material
