# Supplementary material for: Concurrent analysis of hospital stay durations and mortality of emerging severe acute respiratory coronavirus virus 2 (SARS-CoV-2) variants using real-time electronic health record data at a large German university hospital
Source: Antimicrob Steward Healthc Epidemiol. 2023 May 4;3(1):e88. doi: 10.1017/ash.2023.153 (PMC10173280; doi:10.1017/ash.2023.153)
Supplement: Supplementary file 1 [file S2732494X23001535sup001.pdf]

Supplement 1

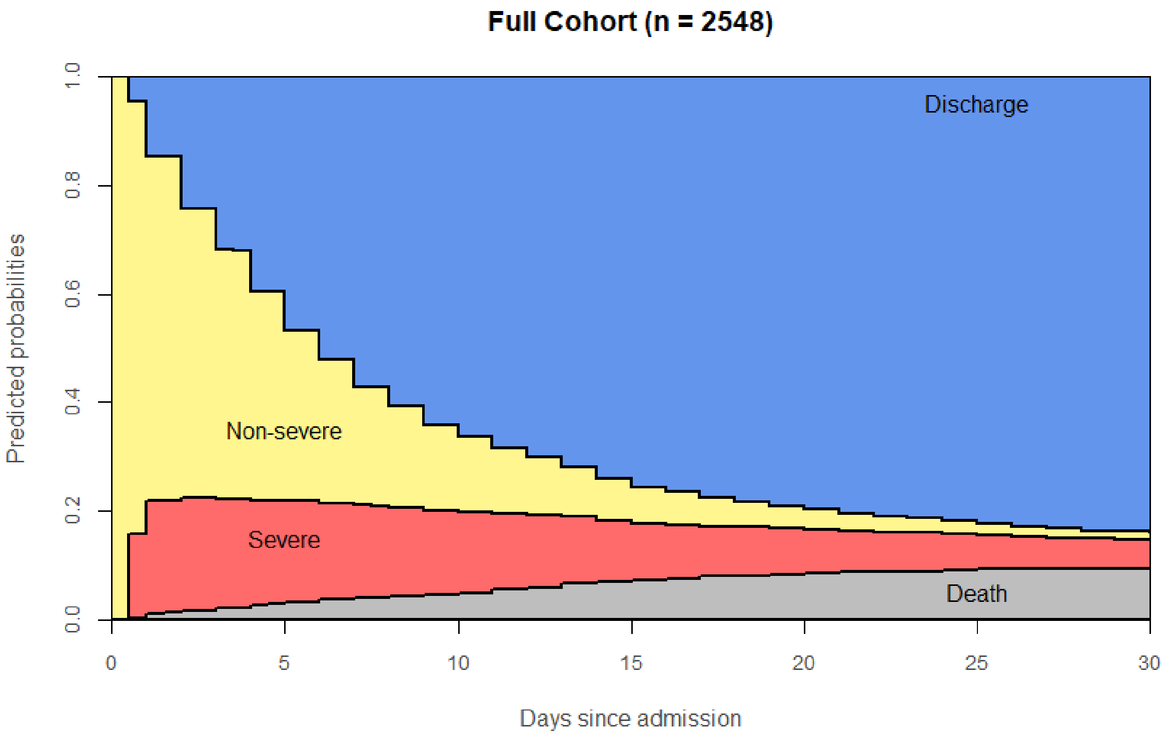

Stacked probability plot for full cohort of 2548 COVID-19 admissions to the University Medical Center Freiburg, Germany. Distances between lines represent proportion of patients in a particular state on day since hospital admission. Discharge: discharged alive from hospital. Non-severe: normal hospital ward. Severe: intensive care unit. Death: death in hospital.

Supplement 2

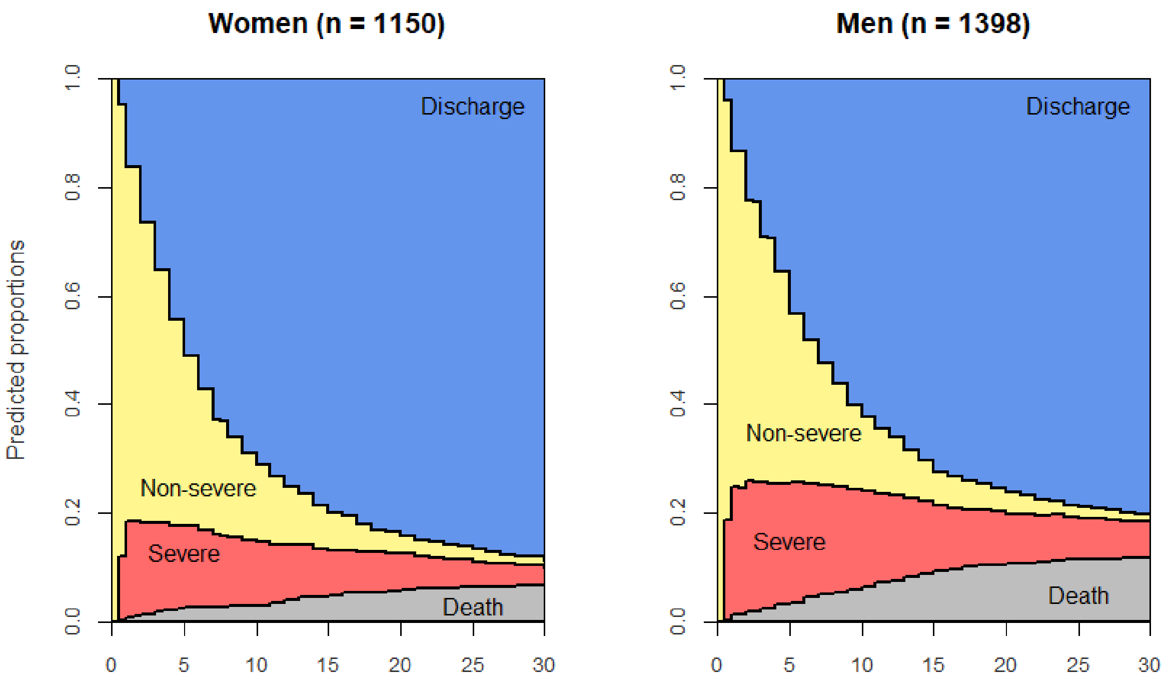

Stacked probability plots stratified by sex of 2548 COVID-19 admissions to the University Medical Center Freiburg, Germany. Distances between lines represent proportion of patients in a particular state on day since hospital admission. Discharge: discharged from hospital. Non-severe: normal hospital ward. Severe: intensive care unit. Death: death in hospital.

Supplement 3

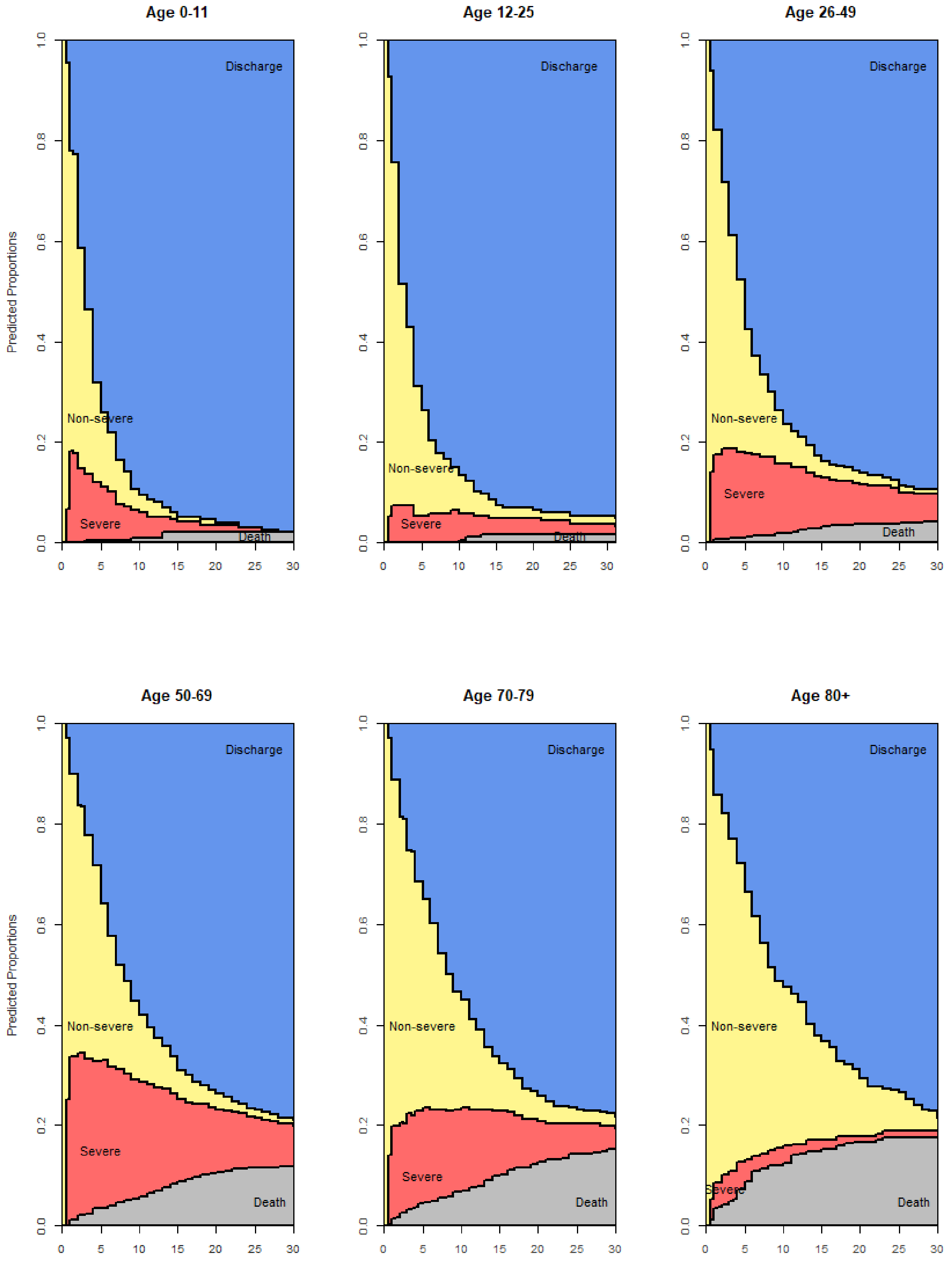

Stacked probability plots stratified by age categories of 2548 COVID-19 admissions from the University Medical Center Freiburg, Germany. Distances between lines represent proportion of patients in a particular state on day since hospital admission. Discharge: discharged alive from hospital. Non-severe: normal hospital ward. Severe: intensive care unit. Death: death in hospital.

Supplement 4

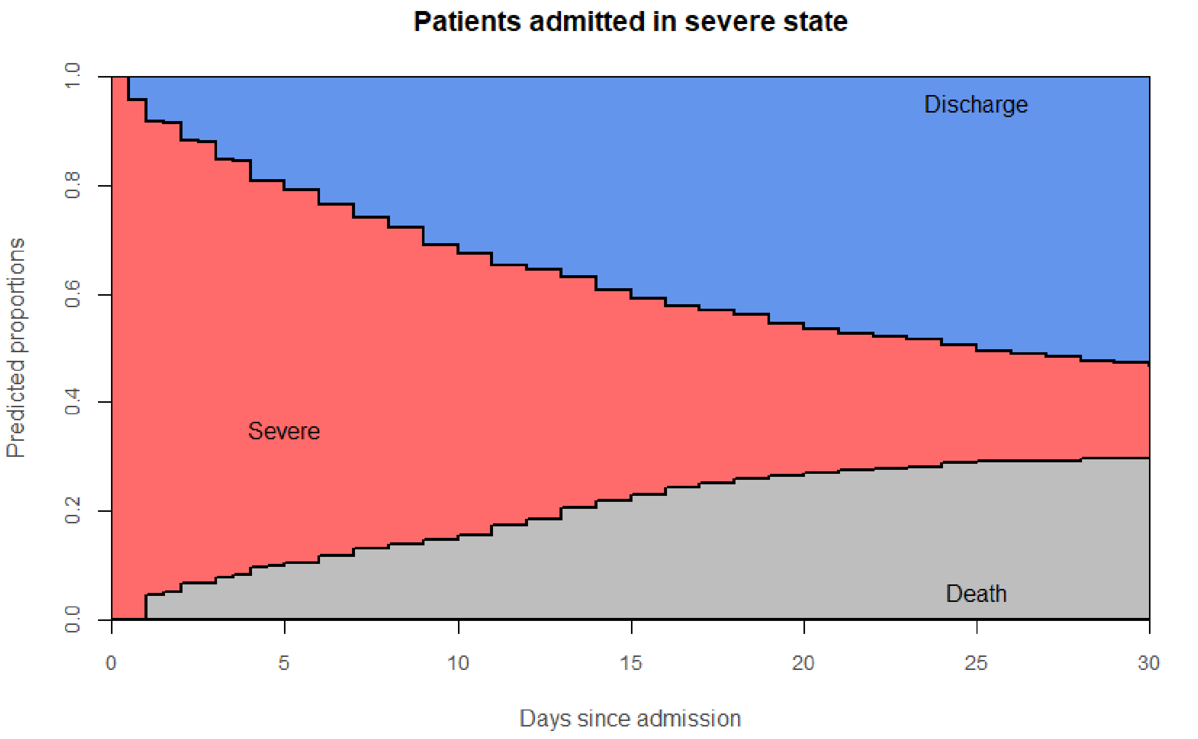

Stacked probability plot for patients admitted into severe state from 2548 COVID-19 admissions from the University Medical Center Freiburg, Germany. Distances between lines represent proportion of patients in a particular state on day since hospital admission. Discharge: discharged alive from hospital. Non-severe: normal hospital ward. Severe: intensive care unit. Death: death in hospital.
